# Atrial Fibrillation and Peri-Atrial Inflammation Measured through Adipose Tissue Attenuation on Cardiac Computed Tomography

**DOI:** 10.3390/diagnostics11112087

**Published:** 2021-11-11

**Authors:** Nicola Gaibazzi, Chiara Martini, Giorgio Benatti, Alessandro Anselmo Palumbo, Giovanna Cacciola, Domenico Tuttolomondo

**Affiliations:** 1 Department of Cardiology, Parma University Hospital, Via Gramsci 14, 43125 Parma, Italy; ngaibazzi@gmail.com (N.G.); gbenatti@ao.pr.it (G.B.); giovanna.cacciola@unipr.it (G.C.); d.tuttolomondo@hotmail.it (D.T.); 2 Section of Radiology, Department of Medicine and Surgery, Parma University Hospital Hospital, Via Gramsci 14, 43125 Parma, Italy; alepalumbo@gmail.com

**Keywords:** atrial fibrillation, adipose tissue attenuation, cardiac computed tomography angiography, inflammation, atrial size

## Abstract

Background: Inflammation plays a key role in atrial fibrillation (AF). Epicardial adipose tissue around the atrial wall can influence atrial morpho-functional properties. The aim of this study was to assess whether an increased quantity and/or density of adipose tissue located around the left atrium (Fat-LA) are related to AF, independently from atrial size. Methods: eighty patients who underwent AF ablation and 80 patients without history of AF were selected. The Fat-LA mass was quantified as tissue within −190 to −30 Hounsfield Units (HU) on cardiac computed tomography angiograms (CCTA), and the mean adipose tissue attenuation was assessed. Results: Adipose tissue mass was higher in patients with AF (5.42 ± 2.94 mL) versus non-AF (4.16 ± 2.55 mL, *p* = 0.007), but relative fat quantity did not differ after adjusting for atrial size. Mean fat density was significantly higher in AF (−69.15 HU) versus non-AF (−76.82 HU, *p* < 0.0001) participants. In the logistic regression models, only the addition of mean Fat-LA attenuation led to a significant improvement of the model’s chi-square (from 22.89 of the clinical model to 31.69 of the clinical and adipose tissue attenuation model, *p* < 0.01) and discrimination (AUC from 0.775 to 0.829). Conclusions: Fat-LA volume is significantly greater only in absolute terms in patients with AF, but this difference does not hold after adjusting for the larger LA of AF subjects. On the contrary, a higher Fat-LA density was associated with AF, independently from LA size, providing incremental value over other variables that are associated with AF.

## 1. Introduction

Several studies have reported an association between epicardial or pericardial adipose tissue and atrial fibrillation (AF) [1,2], a few of them showing a more specific association between the quantity of fat around the atria and AF [3,4,5,6,7,8]. The proposed pathophysiological mechanisms linking epicardial fat with AF include the possible electric remodeling of the atria by adipose tissue infiltration, fibrosis of the adjacent myocardium or indirect mechanisms, such as adipocytes, which represent a source of atrial myocardial inflammation [8,9]. A recent study assessed the mass of the adipose tissue located posterior to the left atrium (Fat-LA), based on the fat density range measured by cardiac computed tomography angiography (CCTA), and concluded that the mass of Fat-LA is significantly higher in AF patients when compared with patients without AF [6]. In any case, fat mass was not corrected for the left atrium (LA) size in the abovementioned study, which does not permit a conclusion as to whether the higher fat mass in AF patients was simply due to a larger atrial size (reverse causality), or that this finding was instead truly, independently associated with the presence of AF. Several studies have indirectly suggested that inflammation is involved in atrial fibrillation, however, direct evidence of local inflammatory activity in the atria of patients with AF is scarce [8,9,10,11,12,13,14]. We hypothesized that the density of adipose tissue, also defined as “attenuation” when measured with CCTA, in close anatomic continuum with the LA, may be considered as a marker of the local inflammatory status, similarly to what has been previously demonstrated for peri-vascular adipose tissue attenuation and vascular inflammation [15,16,17,18,19,20]. The assessment of the mean density of adipose tissue in continuum with the LA will confirm (or contradict) such atrial inflammatory involvement in AF, using in-vivo imaging.

We assessed the Fat-LA for volume and mean attenuation in two groups of patients who underwent CCTA: (I) patients undergoing AF ablation; (II) patients with suspected coronary artery disease (CAD) but without a history of AF. To account for the potential reverse causality bias of larger atria in AF patients (with a higher overall volume sampled) that would drive the finding of a higher absolute quantity of Fat-LA measured in AF patients, we indexed the absolute Fat-LA volume to the LA size and we additionally report adipose tissue percentage within the overall peri-atrial volume sampled, a measure less dependent on the size of the region of interest sampled.

## 2. Materials and Methods

### 2.1. Patients

The current study is a retrospective case–control study. From an ongoing CCTA registry, 80 consecutive patients with AF who were referred for CCTA before radiofrequency catheter ablation (to assess the LA anatomy and location of the pulmonary veins) from January 2015 to January 2019, and 80 patients without AF, who in the same period were referred to CCTA for the evaluation of suspected CAD and who demonstrated no significant CAD (defined as no evidence of luminal plaques or, if present, are associated with less than 30% diameter stenosis), were randomly selected as controls. The presence of AF was defined according to the European Society of Cardiology guidelines for AF management [21]. Contrast-enhanced CCTA data were analyzed for the quantification of the volume of Fat-LA, as well as its mean attenuation, measured in Hounsfield Units (HU). Demographic and clinical data were prospectively collected.

Exclusion criteria: due to a possible interference with inflammatory status we excluded patients with known CAD, prior myocardial infarction in non-obstructive CAD, Takotsubo cardiomyopathy, coronary revascularization or any type of percutaneous intervention, valvular heart disease, aortic or vascular surgery, any active or chronic known inflammatory/infective disease, and known cancer. Finally, if the CCTA was deemed technically not interpretable due to very low quality, these patients were excluded as well.

### 2.2. Coronary Computed Tomography Angiography

Cardiac computed tomography angiography examinations were performed using a Dual Source CTA system (Somatom Definition FLASH, Siemens Healthcare, Forcheim, Germany). To achieve optimal image quality and to reduce the X-ray dose, all patients with a heart rate > 65 bpm received intravenous atenolol 5–10 mg 5 min prior to the CTA scan to reduce motion artifacts. Low and regular heart rates allowed for the scanning of patients using the prospective ECG scan technique or the high-pitch FLASH CTA protocol. All patients received sub-lingual nitrates (isosorbide dinitrate 0.5 mg tablets administered 1–3 min prior to the investigation) to improve visualization of the coronary arteries through dilation.

### 2.3. Fat-LA Volume and Attenuation

**Figure 1:**
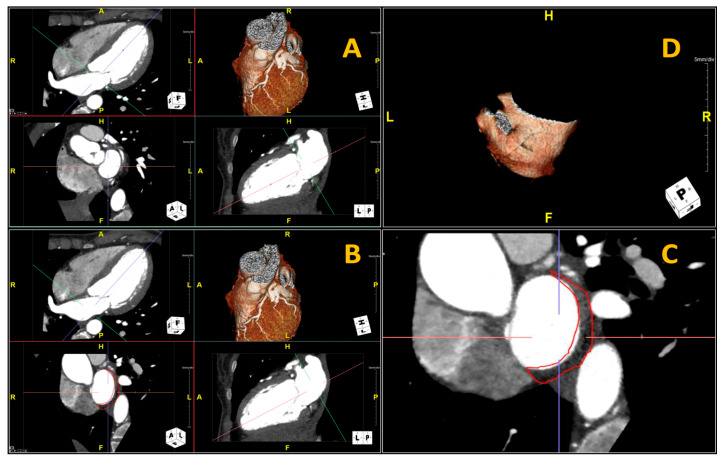
Reconstruction of the cross-sectional view of the left atrium, to measure the adipose tissue located posterior to the left atrium volume and attenuation on the cardiac computed tomography angiogram. (**A**) represents the first slice selected at the coronary sinus position and in (**B**) one slice in the mid-atrial position. (**C**) shows the manually traced region of interest (ROI) of a specific slice. (**D**) shows the reconstruction of three-dimensional ROI.

To measure the Fat-LA volume and attenuation, we used a software package (Aquarius Workstation^®^ V.4.4.13, TeraRecon Inc., Foster City, CA, USA) which allows the tracing of volumetric samples (Figure 1). In this case, standard 2- and 4-chamber views were reconstructed, with slices of 2-mm thickness and the perpendicular plane was set to obtain a cross-sectional view of the LA from the mitral annulus to the LA roof (Figure 1a shows the first slice selected at the coronary sinus position; Figure 1b shows one of the mid-atrial slices). The posterior LA adipose tissue volume was quantified in the short-axis views by manually tracing the pericardium posterior to the LA (Figure 1c). The cranial and caudal limits were respectively defined as the base of the LA (up to the left pulmonary vein insertion) and mitral annulus (coronary sinus, as the marker for the first slice), resulting in ≈20–25 slices depending on the atrial size. The HU of the tissue, within the space demarcated by the posterior LA myocardium and the manually traced pericardium, were automatically determined, and only the tissue within the −190 to −30 HU range (Fat-LA) was defined as adipose tissue (Figure 1d shows the 3D-reconstructed volume of the overall measured adipose tissue); the Fat-LA volume and mean attenuation ± standard deviation (SD) were automatically measured by the software. The Fat-LA attenuation was then weighted for technical parameters; if 100 kV voltage was used instead of the standard 120 kV voltage, the mean HU value was corrected through dividing by 1.11485, as previously reported [22,23].

### 2.4. Statistical Analysis

Continuous variables were presented as the mean ± SD or median and the 25% to 75% interquartile range, according to the distribution. Normally distributed variables were compared with the Student’s t-test. Non-normally distributed variables were compared with the Mann–Whitney U test. Categorical variables were presented as the number and percentage and compared with the χ^2^ test. To assess the independent association of the Fat-LA volume and attenuation with AF, single and multiple variable logistic regression analyses were performed. Odds ratios (OR) and 95% confidence intervals (CI) were derived. Age, gender, body mass index, classic risk factors, main echocardiographic variables (left ventricle ejection fraction, LA size and LA indexed volume), and drug therapies were tested for possible insertion in the clinical model. Only variables with *p* < 0.1 at single variable logistic regression were inserted in the multivariate models. Fat-LA variables (volume and/or attenuation) were added to the starting clinical model. To assess the incremental predictive value of the Fat-LA volume or mean density to the clinical model, (I) chi square for goodness-of-fit, (II) the C-statistic (area under the receiver operating characteristic curve-ROC) for discrimination, (III) the Hosmer-Lemeshow for calibration and (IV) Akaike information criterion for model fitting, were calculated for each model and compared.

A probability value of <0.05 was considered significant. All the statistical analyses were made using Statsdirect software, version 3.0 (Available online: http://www.statsdirect.com (accessed on 11 November 2021) England: StatsDirect Ltd. 2013).

## 3. Results

### 3.1. Patients

Table 1 describes the clinical and echocardiographic characteristics of the, overall, 160 patients included in the study, and the AF versus non-AF (sinus rhythm) groups. The mean age was 58.5 (±12.5) years and 41.9% were female. Patients with AF were significantly older (61.4 ± 10.9 versus 55.5 ± 12.9 years, *p* = 0.004) and were less frequently female (30% *versus* 54%, *p* = 0.002). The median value (lower-upper quartile) of BMI was similar between the two groups (27 (25–30) vs. 25.9 (23.7–28.6) kg/m^2^, *p* = 0.189). In terms of the cardiovascular risk profiles, there were no statistically significant differences regarding the prevalence of diabetes mellitus (3.8 vs. 7.5%, *p* = 0.303), arterial hypertension (61.3 vs. 48.8%, *p* = 0.112), dyslipidemia (36.3 vs. 41.3%, *p* = 0.516), and smokers (43.8 vs. 28.7%, *p* = 0.07). There were no significant differences regarding drug therapy in the two groups. Among the main echocardiographic parameters, the left ventricle ejection fraction was not different between the two groups (60 (55–64) vs. 60 (55–63)%, *p* = 0.723), whereas AF patients had a higher LA area (22.4 (18.3–25.8) versus 18 (15–19.5 cm^2^, *p* < 0.001) and indexed LA volume (37 (33–43) *versus* 33 (23.5–37.5) mL, *p* = 0.030).

### 3.2. Posterior LA Adipose Tissue CTA Volume and Attenuation

The median Fat-LA volume in the overall population was 4.2 (2.6–6.6) mL. The volume of the region of interest (ROI) that was drawn posterior to the left atrium (the overall volume traced to automatically measure the Fat-LA, based on density range) was significantly higher in AF patients when compared with controls, 26.4 (22.4–32.5) versus 18 (14.1–23.6) mL, *p* < 0.001. The absolute measure of Fat-LA volume was significantly higher in AF patients (4.7 (3.2–7.5) versus 3.6 (2.2–5.5) mL, *p* = 0.007), while the percentage of Fat-LA within the overall ROI sampled was not significantly different between the two groups (AF 19.48% ± 9.47 vs. non-AF 21.06% ± 9.54, *p* = 0.317).

**Figure 2:**
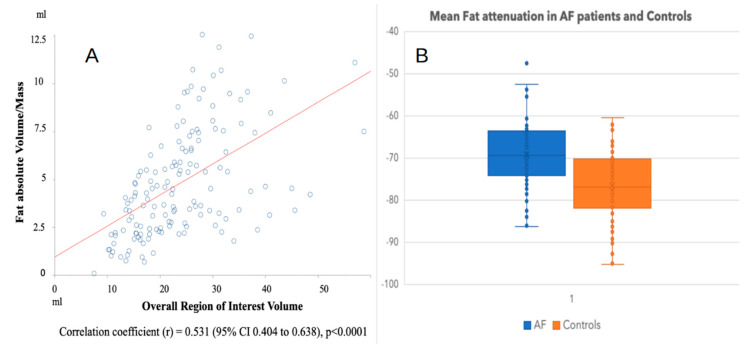
(**A**) The absolute volume of adipose tissue detected that was located posterior to the left atrium was significantly and directly correlated (simple linear regression) with the size of the region of interest sampled. (**B**). Mean adipose tissue (located posterior to the left atrium) attenuation in patients with atrial fibrillation and controls.

As shown in Figure 2a, the absolute Fat-LA volume measured and the overall ROI volume traced were significantly and directly correlated, with *r* = 0.531 (95% CI 0.404 to 0.638), *p* < 0.0001, while the Fat-LA density was instead only mildly correlated with the ROI volume drawn, with *r* = 0.208 (95% CI 0.047 to 0.358) and a borderline significance of *p* = 0.01 (not shown). Patients with AF had significantly higher (closer to 0 HU) values of Fat-LA density when compared with the non-AF group (mean attenuation −69.15 ± 8.28 vs. −76.82 ± 8.54 HU, *p* < 0.001) (Figure 2b). From the ROC curves, the optimum cutoff value for the absolute Fat-LA volume, to separate patients in the group with AF from the ones without AF, was more or equal to 4.22 mL, with an area under the ROC curve of 0.629 (95% CI = 0.539 to 0.719), sensitivity 0.616 (95% CI 0.495 to 0.728), and specificity 0.608 (95% CI 0.488 to 0.720).

The optimum cut-off point that was selected for Fat-LA attenuation was more or less equal to −76.4 HU, with an area under ROC curve = 0.729 (95% CI 0.649 to 0.810), sensitivity (95% CI) = 0.836 (0.730 to 0.912), and specificity (95% CI) = 0.554 (0.434 to 0.670).

### 3.3. Clinical Variables and Fat-LA Volume/Density Logistic Regression Models

Single and multiple variable logistic regression models are reported in Table 2; in the single-variable analysis, both the posterior Fat-LA volume and mean attenuation index were significantly associated with AF; the odds ratio (OR) per milliliter and HU increase were 1.18 (95% CI, 1.04–1.34, *p* = 0.008) and 1.12 (95% CI, 1.07–1.17, *p* < 0.001), respectively. The clinical and echocardiographic model included age, sex, smoker status, and LA area, based on all the demography, risk factors, drug therapy and echocardiographic variables available. Fat-LA volume and/or density were added to this first model. Only the mean attenuation of Fat-LA remained significantly associated with AF (OR 1.09, 95% CI, 1.03–1.11, *p* = 0.006) when added to the clinical and echocardiographic model, whereas the Fat-LA volume did not (OR 1.06, 95% CI, 0.88–1.27), *p* = 0.541. 

### 3.4. Comparison of Logistic Regression Models

**Figure 3:**
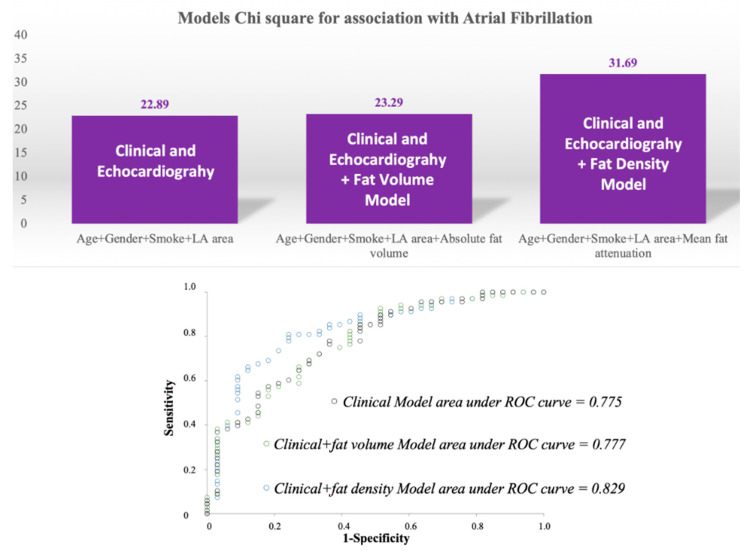
Chi square and area under the ROC curve for the clinical plus echocardiography model compared with the model obtained with the addition of fat volume or fat density variables. LA, left atrium.

The discriminatory ability of the clinical plus the Fat-LA density model, for the prediction of AF (AUC = 0.829), was significantly higher (*p* < 0.001) than the baseline clinical and echocardiography (AUC = 0.775), or clinical and echocardiography plus adipose tissue volume (AUC = 0.777), models. The chi square, as well as the ROC curves and AUC of the models are shown in Figure 3. The clinical model, the clinical plus the Fat-LA volume, and the clinical plus the Fat-LA density models were all well-calibrated, as demonstrated by the Hosmer-Lemeshow test which showed *p* = 0.858, *p* = 0.803, and *p* = 0.740, respectively. The fitting/quality of models was also assessed with the Akaike information criterion, which for the same three models was respectively 114.73, 116.33, 107.94, with the clinical and echocardiography plus Fat-LA attenuation model demonstrating the lowest value, and hence, a higher quality model.

## 4. Discussion

The current study establishes that the mean Fat-LA attenuation is a variable that is independently associated with the presence of AF, and incrementally to other known, associated variables. We hypothesize that this association is due to the fact that a higher Fat-LA attenuation may represent a measure of atrial inflammation, similarly to the established link between peri-vascular fat attenuation and inflammation in the coronary arteries [15,18,22,23]. Fat-LA volume was only apparently associated with AF, since, importantly, it was no longer significant when appropriately accounting for a higher volume of the LA in AF patients, which made their sampled ROI bigger, and their fat measured more abundant; in absolute terms, in conclusion, we found more Fat-LA in AF patients simply because we sampled a larger volume. A prior study which used an appropriate volumetric measurement of Fat-LA as we did (and not the area on a single image) reported that a higher Fat-LA volume is present in AF patients, but the authors did not correct for LA size and, possibly because of this “omitted variable” bias, concluded that Fat-LA volume was associated with AF [6].

### 4.1. State of the Art

The current study is not the first or only study to suggest that atrial inflammation is associated with AF. There is abundant but “indirect” published evidence linking inflammation to AF, mainly based on systemic levels of C-reactive protein, soluble pro-inflammatory cytokines, or “reverse” evidence of a reduced incidence of AF in patients who have been treated with Colchicine, an anti-inflammatory drug [24]. Few studies have also tried to directly and specifically “image” atrial inflammation, as we also did, in our case by taking advantage of the measurement of CCTA adipose tissue attenuation. Most of such “atrial inflammation” imaging data come from isolated case-reports, but also from a few ^18^FDG-PET studies that have demonstrated a higher atrial tracer uptake in AF patients [25,26], although at least one study did not confirm this finding [27]. Furthermore, this metabolic ^18^FDG approach to inflammation imaging is nowadays seriously questioned, as ^18^FDG uptake is probably not truly selective for inflammation or macrophage activity; another criticism concerning the use of ^18^FDG imaging in AF is that, even if it were specific for inflammation, ^18^FDG activity could be weakened by the presence of atrial fibrosis, which is typical of AF, which represents a potential confounder, since it may diminish cumulative atrial cells’ metabolism, and hence ^18^FDG uptake [28].

The assessment of peri-atrial fat density/attenuation using CCTA imaging, a recent method which has been translated from vascular inflammatory assessments, would be a convenient method to image inflammation in the atria, due to the higher spatial resolution of CCTA, lower cost and radiation burden and, importantly, being independent from patient preparation protocols.

Most studies have focused on the quantity of epicardial adipose tissue that is assessed in the heart as a whole, or more specifically assessed around, or posterior to, the atria. This adipose tissue volume, mass, or quantity has generally been either too simplistically assessed, as a one-dimensional measure of “thickness” or a two-dimensional “area” measure (for example in four- or two-chamber equivalent views), or measured as absolute mass/volume in a 3D posterior LA sample, as we also performed in our study [6,29]. We additionally applied the measurement of mean CCTA adipose tissue attenuation to this volumetric Fat-LA sample, something that was never studied before in a case–control study. Higher density/attenuation of Fat-LA in the presence of AF has been, in fact, reported by a recent study, but, importantly, this was as the mean attenuation of a sampled adipose tissue area, a single-plane equivalent view, which limited the reliability of such a measurement, where in fact in one view (four-chamber) fat attenuation was significantly associated with AF, while in the other (two-chamber) it was not [30]. A single 2D-based measure of adipose tissue attenuation is probably unreliable as a marker of attenuation in a 3D volume of tissue. 

The current study used the volumetric sample method, which was validated in recent studies for the measurement of attenuation in the peri-coronary fat, both for volume and attenuation assessments, using the software most widely used for this fat attenuation measurement [15,23].

### 4.2. Fat Attenuation Versus Volume

Adipose tissue attenuation is more reliably accurately measured than fat volume, since it does not rely on any anatomical marker to be manually and subjectively established by the investigator performing the measurement, and whatever the quantity/volume measured, it varies little. The reproducibility of adipose tissue attenuation measurements is very high, while this may not be the case for fat volume, which relies on many subjective issues, such as the number of slices considered by one or the other operator—mainly which is the first and last slice to be used [17,23]. Importantly when Fat-LA was normalized to the left atrial area (or volume), there was no significant difference between the group with AF and those who did not [31]. This seems to clearly contradict a causal relationship between the quantity of adipose tissue and AF, a higher fat mass rather appearing more dependent on the presence of bigger atria, which leads to a higher volume of peri-atrial space sampled. In any case, it should be noted that bias may exist in retrospective studies, and this may pertain, in particular, to the selection of the control group and uncontrolled variables [32,33].

## 5. Conclusions

Peri-atrial inflammation, measured through adipose tissue attenuation on CCTA, was associated with AF independently from the LA size.

These findings add new in-vivo data regarding peri-atrial adipose tissue attenuation, a variable that was non-invasively acquired from standard CCTA, and supporting the hypothesis that local atrial inflammation is truly associated with the presence of AF.

## Figures and Tables

**Table 1:** Data of the demography, risk factors, echocardiography and adipose tissue located posterior to the left atrium.

	Total (*n* = 160)	Atrial Fibrillation (*n* = 80)	Sinus Rhythm (*n* = 80)	*p*-Value
Age, years, mean, SD	58.5 ± 12.5	61.4 ± 10.9	55.5 ± 12.9	0.004
Female gender, *n* (%)	67 (41.9)	24 (30)	43 (54)	0.002
*Cardiovascular risk factors*				
BMI, median, [lower-upper quartile]	26.1 (24.1–29.2)	27 (25–30)	25.9 (23.7–28.6)	0.189
DM, *n* (%)	9 (5.6)	3 (3.8)	6 (7.5)	0.303
HT, *n* (%)	88 (55)	49 (61.3)	39 (48.8)	0.112
Dyslipidemia, *n* (%)	62 (38.8)	29 (36.3)	33 (41.3)	0.516
Smoker, *n* (%)	53 (33.1)	35 (43.8)	23 (28.7)	0.070
*Medications*				
Beta-blocker, *n* (%)	78 (48.8)	44 (55)	34 (42.5)	0.114
ACE-I/ARBs, *n* (%)	72 (45)	41 (51.3)	31 (38.8)	0.112
Statin, *n* (%)	41 (25.6)	18 (22.5)	23 (28.8)	0.365
Ca-antagonist, *n* (%)	18 (11.3)	11 (13.8)	7 (8.8)	0.317
*Echocardiography*				
LVEF, %, median, [lower-upper quartile]	60 (55–64)	60 (55–64)	60 (55–63)	0.723
LA area, cm^2^, median, [lower-upper quartile]	20 (17.2–24)	22.4 (18.3–26)	18 (15–19.5)	<0.001
LAVi, mL/m^2^, median, [lower-upper quartile]	34.1 (27–39)	37 (33–43)	33 (23.5–37.5)	0.030
*Adipose tissue located posterior to the left atrium data on cardiac computed tomography angiography*				
Fat-LA volume, mL, median, [lower-upper quartile]	4.2 (2.6–6.6)	4.7 (3.2–7.5)	3.6 (2.2–5.5)	0.007
ROI volume, mL, median, [lower-upper quartile]	22.7 (16.7–28.1)	26.4 (22.4–32.5)	18 (14.1–23.6)	<0.001
Fat-LA mass relative to ROI, %, median, [lower-upper quartile]	19.8 (12.3–27.1)	19.1 (11.8–26)	21.3 (12.7–28.2)	0.317
Fat-LA attenuation, HU, SD	−73 ± 9.23	−76.82 ± 8.54	−69.15 ± 8.28	<0.001

BMI, body mass index; DM, diabetes mellitus; HT, arterial hypertension; ACE-I, angiotensin-converting enzyme inhibitors; ARBs, angiotensin receptor blockers; LVEF, left ventricle ejection fraction; LA, left atrium; LAVi, left atrium volume index; Fat-LA, adipose tissue located posterior to the left atrium; ROI, region of interest.

**Table 2:** Single and multiple variable logistic regression models.

	Single Model	*p*-Value	Clinical and Echocardiography Model	*p*-Value	Clinical and Fat-LA Volume Model	*p*-Value	Clinical and Fat-LA Density Model	*p*-Value
	*Univariable* *Odds Ratio* *(95% CI)*		*Multivariable* *Odds Ratio* *(95% CI)*		*Multivariable* *Odds Ratio* *(95% CI)*		*Multivariable* *Odds Ratio* *(95% CI)*	
Age	1.04 (1.01–1.07)	0.005	1.02 (0.98–1.06)	0.310	1.02 (0.98–1.06)	0.381	1.03 (0.99–1.07)	0.193
Female gender	0.35 (0.18–0.68)	0.002	0.25 (0.18–1.40)	0.189	0.54 (0.19–1.56)	0.257	0.48 (0.16–1.41)	0.184
*Cardiovascular risk factors*								
BMI	1.02 (0.98–1.09)	0.633	–					
DM	0.59 (0.14–2.57)	0.483	–	–	–	–	–	–
HT	1.70 (0.88–3.27)	0.114	–	–	–	–	–	–
Dyslipidemia	0.73 (0.37–1.41)	0.345	–	–	–	–	–	–
Smoker	2.54 (1.36–4.74)	0.004	1.24 (0.52–2.94)	0.624	1.25 (0.52–2.97)	0.614	1.02 (0.41–2.51)	0.972
CHA_2_DS_2_-VASc score	0.91 (0.70–1.18)	0.458	–	–				
*Medications*								
ACE-I/ARB	1.65 (0.83–3.31)	0.156	–	–	–	–	–	–
Beta-blocker	1.70 (0.85–3.38)	0.133	–	–	–	–	–	–
Statin	0.65 (0.30–1.43)	0.289	–	–	–	–	–	–
*Echocardiography*								
LVEF	1.02 (0.97–1.07)	0.471	–	–	–	–	–	–
LA area	1.24 (1.11–1.38)	<0.001	1.18 (1.06–1.33)	0.004	1.19 (1.06–1.34)	0.004	1.17 (1.03–1.32)	0.015
*Adipose tissue located posterior to the left atrium on cardiac computed tomography angiography*								
Fat-LA volume	1.18 (1.04–1.34)	0.008			1.06 (0.88–1.27)	0.541	–	–
Fat-LA mean attenuation	1.12 (1.07–1.17)	<0.001			–	–	1.09 (1.03–1.11)	0.006

BMI, body mass index; DM, diabetes mellitus; HT, arterial hypertension; ACE-I, angiotensin-converting enzyme inhibitors; ARBs, angiotensin receptor blockers; LVEF, left ventricle ejection fraction; LA, left atrium; Fat-LA, adipose tissue located posterior to the left atrium.

## Data Availability

Available upon request.
